# The Metabaging Cycle

**DOI:** 10.1111/cpr.13197

**Published:** 2022-02-02

**Authors:** Shilin Ma, Ng Shyh‐Chang

**Affiliations:** ^1^ State Key Laboratory of Stem Cell and Reproductive Biology Chinese Academy of Sciences Beijing China; ^2^ Institute for Stem Cell and Regeneration Chinese Academy of Sciences Beijing China; ^3^ University of Chinese Academy of Sciences Beijing China; ^4^ 74519 Beijing Institute for Stem Cell and Regenerative Medicine Beijing China

## CONFLICT OF INTEREST

All authors declare that they have no conflict of interest.

## AUTHOR CONTRIBUTIONS

SM and NS‐C designed and wrote the manuscript.

To the editor,

The metabolic health of adipose tissue and skeletal muscle is critically intertwined with the overall health of the human body. Muscle and adipose tissues’ functional decline will bring a series of metabolic and health problems with aging, including adipose and muscle inflammatory infiltration and insulin resistance (IR), ultimately leading to the metabolic syndrome (MetS). Accumulating evidence suggests that the balance of adipose tissue and muscle metabolism is essential for health.

Adipose tissue redistribution and ectopic fat deposition, which involves subcutaneous adipose tissue degenerative atrophy and inflammation, visceral adipose tissue (VAT) expansion and exhaustion and lipid infiltration into visceral organs (e.g., NAFLD) and muscles (e.g., myosteatosis), occur during the course of obesity.^1^ Obesity, or adipose tissue expansion, is not directly indicative of metabolic dysfunction per se. Obesity implies an adaptive response to overnutrition in a healthy body, whereby adipocytes store excess lipids, also partly to avoid lipotoxicity to other tissues.^2^ This beneficial metabolic process is compromised during aging or persistent overnutrition, when subcutaneous, and then visceral adipocytes successively reach their limits^3^ and undergo inflammation, exhaustion, atrophy and senescence, which will result in central obesity and hyperlipidemia.^4^, ^5^, ^6^ In addition, the secretome of senescent adipocytes aggravates the metabolic disorder, and immune cells around adipocytes will accelerate secretion of pro‐inflammatory factors such as TNF‐α and the interleukins, thus worsening the adipose tissue inflammation. Inflammation will promote IR, resulting in more lipolysis and accelerated hyperlipidemia.^7^, ^8^, ^9^

Excessive hyperlipidemia will upregulate fatty acid oxidation (FAO) in myocytes and fibro–adipogenic progenitor (FAP) cells, the intermediates and side products of which can regulate cell fate by regulating epigenetic modifications,^10^, ^11^ thus influencing them to differentiate into adipocytes or myofibroblasts, impairing muscle regeneration and aggravating muscle dysfunction. Hyperlipidemia also leads to muscle lipid infiltration, which not only results in myosteatosis in the form of intramyocellular lipid (IMCL) droplets and intermuscular adipose tissue (IMAT), but also overloads the skeletal muscle mitochondria.^12^ Mitochondrial degeneration with myocyte aging aggravates the damage of lipid infiltration, as the secondary products of lipid metabolism such as ceramides and reactive oxygen species (ROS) accumulate with increasing mitochondrial dysfunction.^13^, ^14^ All these factors will impair insulin‐PI3K‐mTOR signalling and promote inflammatory signalling, which in turn crosstalk to result in muscle IR and muscle atrophy or sarcopenia.^15^, ^16^ Muscle IR decreases glucose and lipid uptake, decelerates muscle anabolic growth, accelerates senescence^17^ and exacerbates systemic hyperglycemia, hyperlipidemia and hyperinsulinemia. Degenerating or senescent myocytes will also secrete a variety of pro‐inflammatory factors and chemokines, activating and recruiting a variety of inflammatory cells like macrophages to trigger more complex immune responses, thereby worsening muscle inflammation.^18^ Myocyte inflammation could lead to myocyte apoptosis, muscle proteolysis and fibrosis, all of which will accelerate sarcopenia.^19^

**FIGURE 1:**
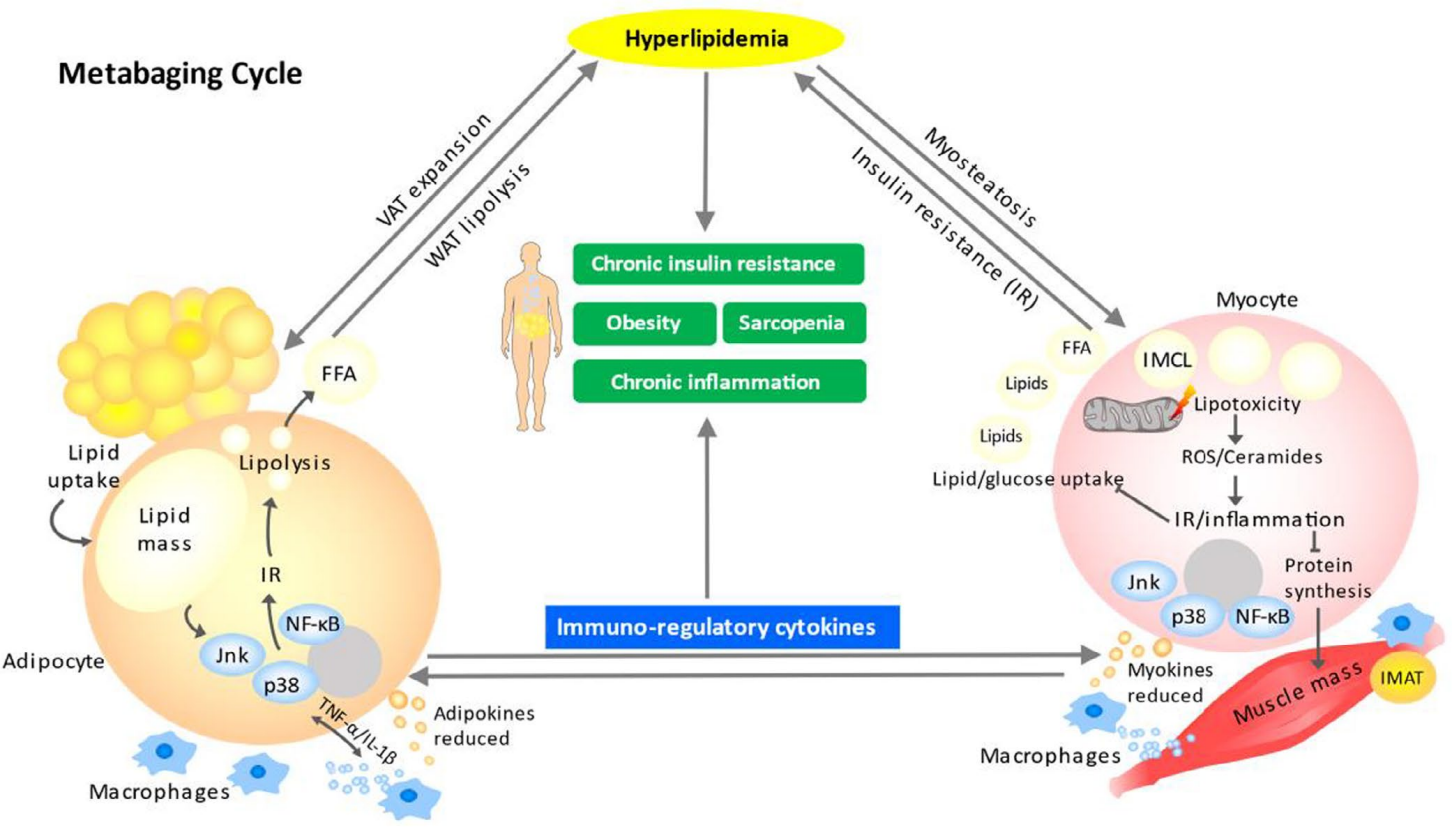
The Metabaging Cycle. The visceral adipose tissue (VAT) expands when hyperlipidemia occurs, but excessive hyperlipidemia will lead to over‐accumulation of lipid mass, adipocyte inflammation and adipocyte senescence, which reduces adipokines, induces insulin resistance (IR) and lipolysis and alters immunoregulatory cytokine secretion. Hyperlipidemia also leads to myosteatosis, inducing lipid infiltration in skeletal muscle through the formation of intramyocellular lipid droplets (IMCL) and intermuscular adipose tissue (IMAT), thereby aggravating lipotoxicity‐induced mitochondrial dysfunction, reactive oxygen species (ROS), ceramides and IR‐inflammatory signalling to NF‐κB, p38 and Jnk, resulting in a suppression of protein synthesis and muscle mass, changes in immune‐regulatory cytokines, reduced myokines and local inflammation. The inflammatory crosstalk between adipose and muscle tissues creates a two‐way vicious cycle which exacerbates both the hyperlipidemia and immune dysregulation, eventually leading to chronic insulin resistance and chronic inflammation, resulting in obesity and sarcopenia

At the physiological level, obesity and sarcopenia often coincide during aging,^20^, ^21^ and at the cellular level, lipotoxicity often coincides with myocyte and adipose inflammation.^22^, ^23^ In this process, adipose tissue and skeletal muscle could mutually influence each other by secreting pro‐inflammatory factors, leading to a vicious cycle of metabolic impairment and further inflammation, which further spreads and progresses to systemic inflammation and IR.^24^, ^25^, ^26^ Here, we name this concept the ‘Metabaging Cycle’ to represent how local adipose exhaustion, inflammation and local hyperlipidemia can cause local myosteatosis and local muscle IR, which in turn leads to chronic systemic inflammation and hyperlipidemia in a two‐way vicious cycle (Figure 1). As a result of the lipid redistribution, the body progresses from incipient obesity to central obesity, and although significant changes in net body weight might not occur at this stage, muscle mass and function are already declining. As the adipocyte/myocyte inflammation and IR continue to spread and worsen, the two tissues’ interplay will lead to a lipotoxic vicious cycle that ultimately causes systemic IR and a variety of MetS‐related chronic diseases, including obesity‐related diseases and sarcopenia, culminating in an impairment in health and longevity (Figure 1).

Aging is the net result of a functional decline in various organs and tissues, and systemic IR/inflammation‐induced senescence is an important factor that triggers this deterioration. Once the vicious cycle of Metabaging occurs, it will become increasingly difficult to reverse, because the interactions between multiple organs and tissue systems make the systemic IR/inflammation situation increasingly more complicated and interlinked. Regular exercise could help stimulate and maintain mitochondrial homeostasis through mitohormesis, so that myocytes which make up ~40% of our body mass can counter and resist the lipotoxicity more avidly, and also increase their secretion of beneficial myokines,^27^ thereby maintaining muscle function and slowing down the vicious cycles of systemic IR/inflammation that drive Metabaging. With our theory on the Metabaging Cycle, and the well‐known phenomenon of low‐grade chronic inflammation during aging, we believe there is no such thing as normal physiological aging. We believe that all aging is rooted in pathological dysfunction, and both chronic low‐grade inflammation and Metabaging gradually rise with age and an increase in metabolic/inflammatory overload due to the mammalian lifestyle. Thus, the lack of visible pathological disease in ‘normal’ aging merely means that the chronic low‐grade inflammation and Metabaging have not crossed the thresholds to manifest in any organ/tissue as a disease. Medication, such as some reported to show benefits to muscle metabolism and countering systemic IR and inflammation, including metformin and pioglitazone, might also help prevent the crossing of disease thresholds and achieve an improved balance in skeletal muscle and adipose tissue metabolism to slow down the Metabaging Cycle, thereby extending human healthspan.^28^

## Data Availability

The authors declare that all the data supporting the findings of this study are available within the article and from the corresponding authors upon reasonable request.
